# Ultrasensitive Electrochemical Immunoassay for Avian Influenza Subtype H5 Using Nanocomposite

**DOI:** 10.1371/journal.pone.0094685

**Published:** 2014-04-14

**Authors:** Zhixun Xie, Jiaoling Huang, Sisi Luo, Zhiqin Xie, Liji Xie, Jiabo Liu, Yaoshan Pang, Xianwen Deng, Qing Fan

**Affiliations:** Guangxi Key Laboratory of Animal Vaccines and Diagnostics, Guangxi Veterinary Research Institute, Nanning, Guangxi Province, China; Boston University School of Medicine, United States of America

## Abstract

We report a novel electrochemical immunosensor that can sensitively detect avian influenza virus H5 subtype (AIV H5) captured by graphene oxide-H5-polychonal antibodies-bovine serum albumin (GO-PAb-BSA) nanocomposite. The graphene oxide (GO) carried H5-polychonal antibody (PAb) were used as signal amplification materials. Upon signal amplification, the immunosensor showed a 256-fold increase in detection sensitivity compared to the immunosensor without GO-PAb-BSA. We designed a PAb labeling GO strategy and signal amplification procedure that allow ultrasensitive and selective detection of AIV H5. The established method responded to 2^−15^ HA unit/50 µL H5, with a linear calibration range from 2^−15^ to 2^−8^ HA unit/50 µL. In summary, we demonstrated that the immunosenser has a high specificity and sensitivity for AIV H5, and the established assay could be potentially applied in the rapid detection of other pathogenic microorganisms.

## Introduction

H5N1 influenza virus is highly pathogenic in poultry, wild birds, and has occasionally infected humans with serious and fatal outcomes [1]. Since 2003, the WHO has reported H5N1 in more than 46 countries for animal cases and 15 countries for human cases with 650 people infected and 386 dead [2]. A variety of technologies for diagnosing avian influenza virus (AIV) have been developed, such as virus isolation, serologic assays, enzyme-linked immunosorbent assay, and polymerase chain reaction (PCR)-based assays [3]–[9]. However, there are some disadvantages with these diagnostic methods making them less ideal in practical applications. For example, these methods either poor in specificity, low in sensitivity, time consuming, or requiring a well equipped laboratory and highly trained technicians [10]–[12].

Electrochemical immunosensors are particularly attractive due to their high sensitivity, capacity for quick analysis, easy for pretreatment, small analyte volume, simple instrumentation, minimal manipulation and wide range of uses [13]–[15]. Several electrochemical immunosensors have been developed and extensively applied to detect antigens [16]–[17]. In order to meet the increasing demand for early and ultrasensitive detection of biomarkers, various signal amplification technologies using nanomaterials have been developed [18]–[20]. Graphene oxide (GO) monolayers made from carbon atoms packed into dense honeycomb crystal structures, have unique nanostructures and properties that render them suitable as electrochemical biosensors. For example, they are in good colloidal condition, have a large surface area and their manufacturing costs are low [21]–[22]. Our present work is motivated by the promising applications of BSA functionalized GO in signal amplification for ultrasensitive detection of AIV H5.

## Materials and Methods

### Avian pathogens and culture conditions

The avian pathogens used in this study are listed in Table 1. Inactivated H5N1 was provided by the Harbin Veterinary Research Institute, China. Inactivated H5N2, H5N9, H7N2 were provided by the Pennsylvania State University, US. Aside from the H5 and H7 subtypes, all other AIV subtypes, newcastle disease virus (NDV), and infectious bronchitis virus (IBV), respectively were propagated in the allantoic cavity of 10-day-old specific-pathogen-free (SPF) embryonated chicken eggs, whereas infectious laryngotracheitis virus (ILT) was propagated on the chorioallantoic membrane in 10-day-old SPF embryonated chicken eggs as described elsewhere [4], [23]. The allantoic fluids from embryonated eggs infected with AIV, NDV, ILT and IBV were harvested after incubation at 37°C for 36 h [4], [23]. *Mycoplasma gallisepticum* (MG) was propagated in Frey's broth and incubated at 37°C as previously described [24].H5-polychonal antibodies and H5-monoclonal antibodies were purchased from Abcam (Cambridge, UK). Graphite powder (<45 mm), chloroauric acid (HAuCl_4_), 1-ethyl-3-(3-dimethylaminopropyl) carbodiimide hydrochloride (EDC), sodium chloroacetate (ClCH_2_COONa), bovine serum albumin (BSA, 96–99%), N-hydroxysuccinimide-activated hexa-(ethylene glycol) undecane thiol (NHS) were all acquired from Sigma-Aldrich. All other reagents were of analytical reagent grade and used without further purification. Phosphate buffered solution (PBS; 10 mmol·L^−1^), at various pH values were prepared by mixing stock solutions of NaH_2_PO_4_ and Na_2_HPO_4_.

**Table 1:** Sources of pathogens used and electrochemical immunoassay assay results.

Avian pathogen samples	Source	H5 electrochemical immunoassay
Inactivated H5N1 AIV Re-1	HVRI	+
Inactivated H5N2/chicken/QT35/87	PU	+
Inactivated H5N9/chicken/QT35/98	PU	+
H1N3 AIV Duck/HK/717/79-d1	HKU	−
H2N3 AIV Duck/HK/77/76	HKU	−
H3N6 AIV Duck/HK/526/79/2B	HKU	−
H4N5 AIV Duck/HK/668/79	HKU	−
H6N8 AIV Duck/HK/531/79	HKU	−
Inactivated H7N2/chicken PA/3979/97	PU	−
H8N4 AIV Turkey/ont/6118/68	HKU	−
H9N6/Duck/HK/147/77	HKU	−
H10N3 AIV Duck/HK/876/80	HKU	−
H11N3 AIV Duck/HK/661/79	HKU	−
H12N5 AIV Duck/HK/862/80	HKU	−
H13N5 AIV Gull/MD/704/77	HKU	−
NDV	GVRI	−
IBV	GVRI	−
ILTV	GVRI	−
MG	GVRI	−
Liver, lung and small intestine of SPF chicken		−

HVRI = Harbin Veterinary Research Institute, China.

HKU = The University of Hongkong, China.

GVRI = Guangxi Veterinary Research Institute, China.

PU = Pennsylvania State University, USA.

### Instruments

For electrochemical studies, we employed a CHI660D electrochemical workstation (Shanghai CH Instruments, Shanghai, China) with a standard three-electrode cell that contained a platinum wire auxiliary electrode, a saturated calomel reference electrode (SCE) and a working electrode (the modified electrode as working electrode). All potential values given refer to SCE. All experiments were performed at room temperature (25±0.5°C).

### Synthesis of graphene oxide

Graphene oxide (GO) was prepared by modified Hummers method [25]. Typically, 1.0 g of graphite powder and 2.5 g of NaNO_3_ were added to 100 mL of concentrated H_2_SO_4_ and stirred for 1 h. The mixture was continuously stirred and ice-cooled as 5 g of KMnO_4_ was slowly added. The mixed slurry was then stirred at 35°C for 20 h. After that, 100 mL of deionized water was added slowly to the reacted slurry and then stirred at 85°C for another 2 h. Next, 300 mL of deionized water was added to the reacted slurry. Then, 6 mL of 30% H_2_O_2_ was added; the slurry immediately turned into a bright yellow solution and bubbles appeared. The resultant solution was stirred for 2 h and then allowed to precipitate for 24 h; after that, the supernatant was decanted. The resultant yellow slurry was centrifuged and washed with 500 mL of 0.5 mol/L HCl. After stirring for 2 h, the solution was centrifuged, washed again, before further washing with deionized water until the pH of the solution increased to neutral (pH 7.0). The remaining dark-yellow solid was dried under vacuum at 40°C for 48 h and ground to a fine powder. The drying process for GO was conducted at low temperatures because GO slowly decomposes (deoxygenates) above 60−80°C. 1.0 mg of GO fine powder was added into 1 mL of deionized water and stirred for 30 min to obtain 1.0 mg/mL GO aqueous solution. Then 1.0 mg/mL GO aqueous solution was placed into an ice bath and sonicated. The ice bath was changed after each treatment to make sure that sample temperature was below 5°C. Finally, the resultant sample was centrifuged at 12,000 rpm for 10 min; the upper solution was used in the experiments.

### Preparation of GO-PAb-BSA bioconjugates

To convert hydroxyl and epoxide groups to carboxylic groups, 50 mg of NaOH and 50 mg of ClCH_2_COONa was added to 1 mL of a 1 mg/mL GO suspension, which was followed by bath sonication for 1 h. After these treatments, the resulting product, GO-COOH, was neutralized with dilute hydrochloric acid and purified by repeated rinsing and centrifugation until the product was well-dispersed in deionized water. The GO-COOH suspension was then dialyzed against distilled water for over 48 h to remove any ions. For the preparation of GO-PAb-BSA bioconjugates, 400 µL GO (0.1 mg/mL) was activated with 10 µL EDC (5 mg/mL) and 20 µL NHS (3 mg/mL) in PBS buffer (pH 5.2) and activated for 30 min. The mixture was centrifuged at 13,000 rpm for 10 min, and the supernatant was discarded. The buffer wash was repeated to remove excess EDC and NHS. The resulting functionalized mixture was dispersed in 1.0 mL of PBS buffer (pH 7.4) and sonicated for 5 min to obtain a homogeneous suspension. Then, 1 mL of PAb (1 µg/mL) and 2 mL of BSA [0.25% (w/v)] were added to the suspension, and the mixture was stirred overnight at 4°C. The reaction mixture was washed with PBS and centrifuged at 13,000 rpm for 5 min, three times. The supernatant was discarded. The resulting mixture was redispersed in 1.0 mL of PBS (pH 7.4) and stored at 4°C.

### Fabrication of the immunosensor

**Figure 1:**
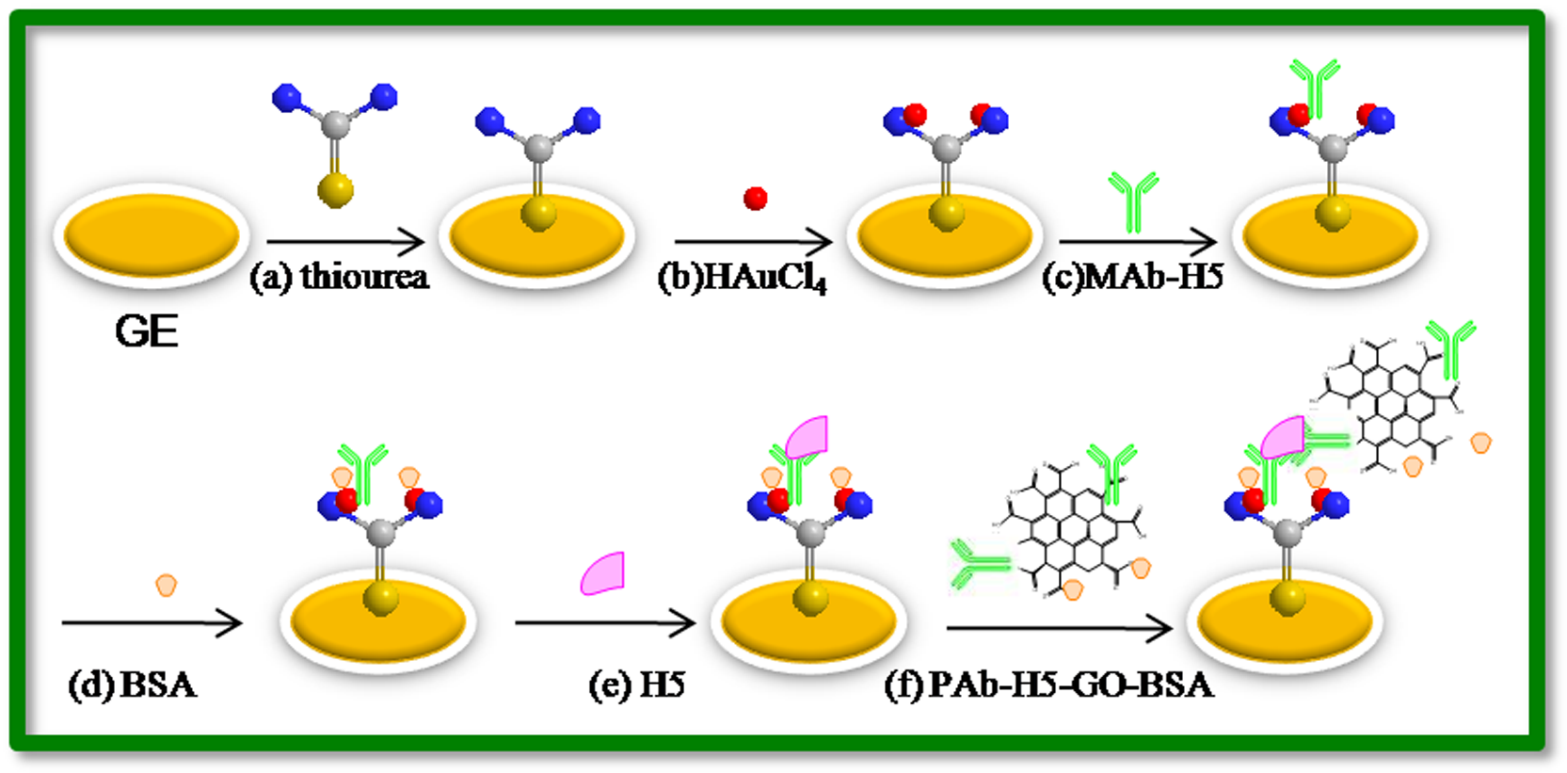
Immunosensor fabrication process: (a) adsorption of thiourca; (b) formation of gold nanoparticles; (c) MAb loading; (d) blocking with BSA; (e) incubation with H5 antigen; (f) GO-PAb-BSA nanocomposite loading.

The gold electrode (GE; Ø = 3 mm) was initially polished with 0.05 mm alumina to obtain a mirror-like surface before being rinsed with distilled water and placed into an ultrasonic bath to remove any physically adsorbed substances. Next, the electrode was placed into an electrochemical cell with 0.05 M H_2_SO_4_ and chemically cleaned until the background signal stabilized. The clean electrode was thoroughly rinsed with ddH_2_O, dried with nitrogen gas, quickly immersed in the 5 mM thiourea solution and incubated at room temperature for 24 h. To prepare the gold nanoparticle-modified surface, a potential scan was applied to the thiourea-gold electrode, which started at 0 V (scan rate of 50 mV·s^−1^) and held at the peak potential −0.2 V for 60 s in a solution of 1% HAuCl_4_. After that, the electrode was washed with ddH_2_O and immersed in PBS solution (pH 7.4) containing 10 µg/mL H5-monoclonal antibodies (MAb), and immobilized at 4°C overnight. Finally, the modified electrode was incubated in 0.25% BSA solution for 1 h at 37°C to block any remaining active sites on the gold nanoparticle (AuNP) monolayer, and thus avoiding non-specific adsorption. The finished immunosensor was stored at 4°C. The procedures used for construction of the immunosensor are shown in Figure 1.

### Immunoassay for detection of H5 antigen

A sandwich immunoassay was used for detection of AIV H5. First, the immunosensor, MAb-AuNPs-thiourea-GE, was incubated with 100 µL of various concentrations of H5 antigen for 30 min, washed with PBS buffer. Next, the electrode was incubated with 200 µL of GO-PAb-BSA bioconjugates for 40 min, washed with PBS buffer to remove non-specific adsorption conjugates. Finally, electrochemical detection was performed in the presence of 5 mM [Fe(CN)_6_]^4−/3−^ and 0.01 M PBS (containing 0.1 M KCl; pH 7.0).

### Criteria for judgement of positive or negative samples

Eighteen negative samples from SPF chickens were measured by the immunosensor, and the average current was calculated. The principle of statistics was used to distinguish positive from negative.

### Performance of immunosensor

Avian influenza virus H5 was diluted to 2^−15^∼2^2^ HA unit/50 µL with PBS, then use immunosensor to test different concentrations of the virus from high to low. All of the experiments were repeated three times, and the average currents of each concentrations were calculated.

### Specificity of immunosensor assay

To evaluate the specificity of the immunosensors, some non-target samples such as H1N3, H2N3, H3N6, H4N5, H6N8, H7N2, H8N4, H9N6, H10N3, H11N3, H12N5, H13N5, NDV, ILTV, IBV and MG were tested by using the developed immunosensors. The immunosensor was incubated with 100 µL samples for 30 min, washed with PBS buffer, then incubated with 200 µL of GO-PAb-BSA bioconjugates for 40 min, and washed with PBS buffer, Lastly, the electrochemical detection was performed in the presence of 5 mM [Fe(CN)6]4–/3– and 0.01 M PBS (containing 0.1 M KCl; pH 7.0). All of the experiments were repeated three times. The H5 antigen was used as a positive control, and tissue from the SPF chickens were used as negative controls.

## Results

### Electrochemical characteristics of different electrodes

**Figure 2:**
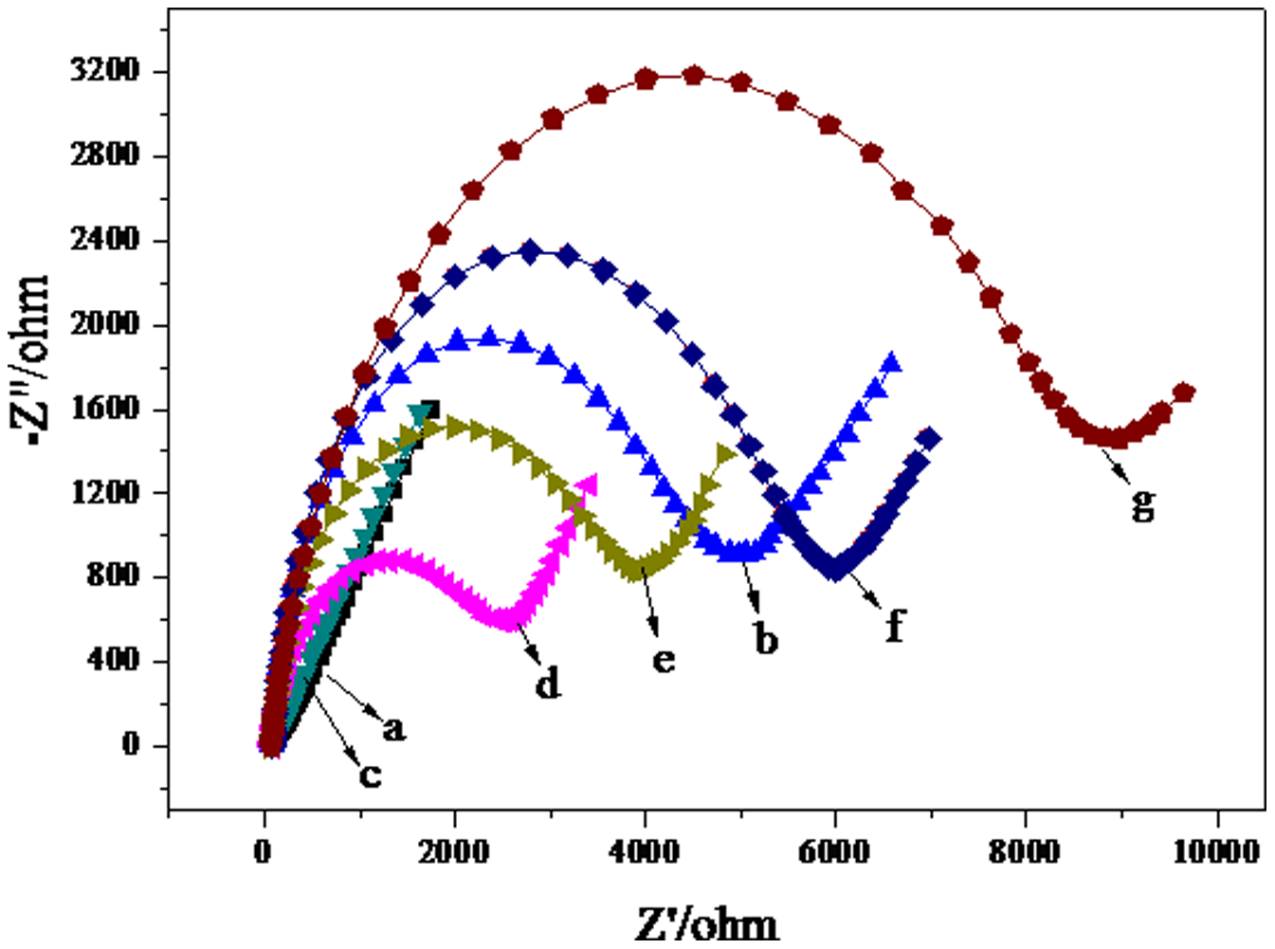
EIS of the different electrodes: (a) bare gold electrode (GE); (b) thiourea-GE; (c) AuNPs–thiourea-GE; (d) MAb-AuNPs–thiourea-GE; (e) BSA-MAb-AuNPs–thiourea-GE; (f) H5-BSA-MAb-AuNPs–thiourea-GE; (g) GO-PAb-BSA-H5-BSA-MAb-AuNPs-thiourea-GE. Supporting electrolyte was 5(CN)_6_^3−/4−^, 0.1 M KCl and 0.01 M PBS (pH 7.0). The frequency range was between 0.1 and 100,000 Hz (AC 5 mV, DC 0.24 V vs. SCE).

Electrochemical impedance spectroscopy (EIS) is regarded as an effective technique for probing the features of surface modified electrodes. The impedance spectrum includes a semicircle portion and a linear portion. The semicircle diameter corresponds to the electron-transfer resistance (R_et_), and the linear part corresponds to the diffusion process. As shown in Figure 2, for the bare GE, we observed a linear part at low frequencies (curve a), suggesting a very low R_et_ to redox probe [Fe(CN)_6_]^3−/4−^. After the bare GE was modified with thiourea, the resistance for the redox probe increased (curve b). R_et_ then decreased when AuNPs were adhered (curve c), proving that AuNPs promote electron transfer and enhance the conductivity of the electrode. Subsequently, when the MAb was loaded on the surface of the AuNPs, the EIS showed a large increase in diameter (curve d), indicating that the antibody forms an additional barrier and further prevents transfer between the redox probe and the electrode surface. The result is consistent with the notion that the hydrophobic layer of protein insulates the conductive support and hinders the interfacial electron transfer. After BSA was used to block non-specific sites, R_et_ increased in a similar manner (curve e), possibly attributed to the same reason as when H5 antigen was loaded. R_et_ increased (curve f) after the resulting immunosensor was incubated in H5 antigen at 2^−4^ HA unit/50 µL, which indicates the formation of hydrophobic immunocomplex layer embarrassing the electron transfer. R_et_ further increased (curve g) after the resulting immunosensor was incubated in GO-PAb-BSA bioconjugates. The impedance change obtained after the modifying process implies that thiourea, MAb, BSA, H5 and GO-PAb-BSA have been assembled successively onto the GE electrode.

**Figure 3:**
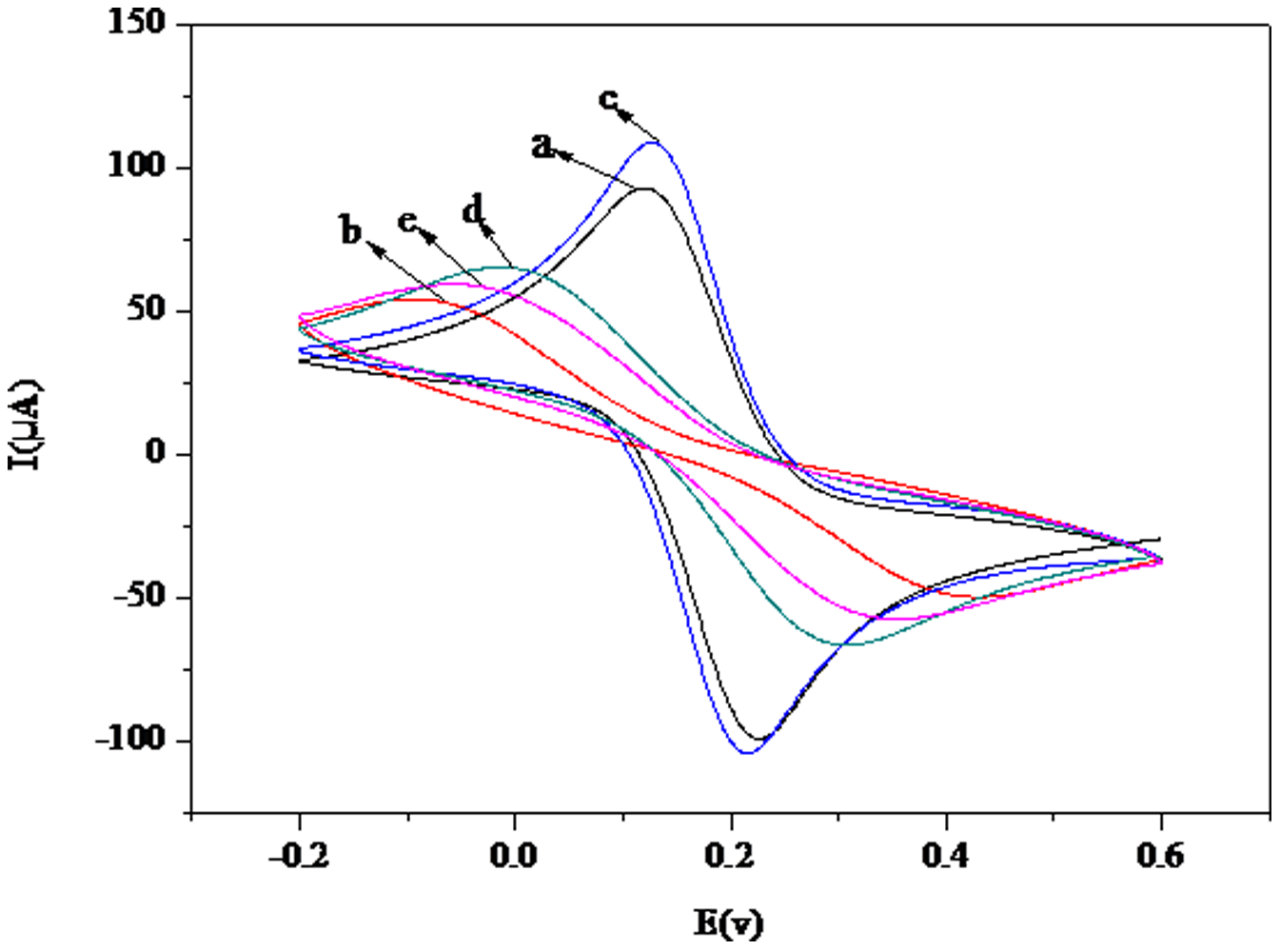
Cyclic voltammograms of the electrode at different stages. Scan rate was 50^−1^. (a) Bare gold electrode (GE); (b) thiourea-GE; (c) AuNPs–thiourea-GE; (d) MAb-AuNPs–thiourea-GE; (e) BSA-MAb-AuNPs–thiourea-GE; (f) H5-BSA-MAb-AuNPs-thiourea-GE. Supporting electrolyte was 5 mM Fe(CN)_6_^3−/4−^, 0.1 M KCl and 0.01 M PBS (pH 7.0).

Cyclic voltammetry (CV) technique was used to study the assembly process of the modified electrode. The CV scans of the different modified electrodes are shown in Figure 3. A well-defined redox wave is shown in curve a, corresponding to the reversible redox reaction of ferricyanide ions on the bare GE electrode. The redox peaks then apparently disappeared after thiourea was coated onto the electrode surface, owing to a thiourea film that greatly obstructed electron and mass transfer (curve b). When the bare GE was modified with AuNPs, the peak current of the system gradually increased (curve c), potentially due to AuNPs effectively increasing the surface area and active sites of the electrode. The immobilization of MAb on the electrode surface resulted in a decreased peak current (Figure 3d), which suggests that MAb severely reduces the surface area and active sites needed for electron transfer. The peak current slightly decreased (Figure 3e) after BSA was used to block non-specific sites.

**Figure 4:**
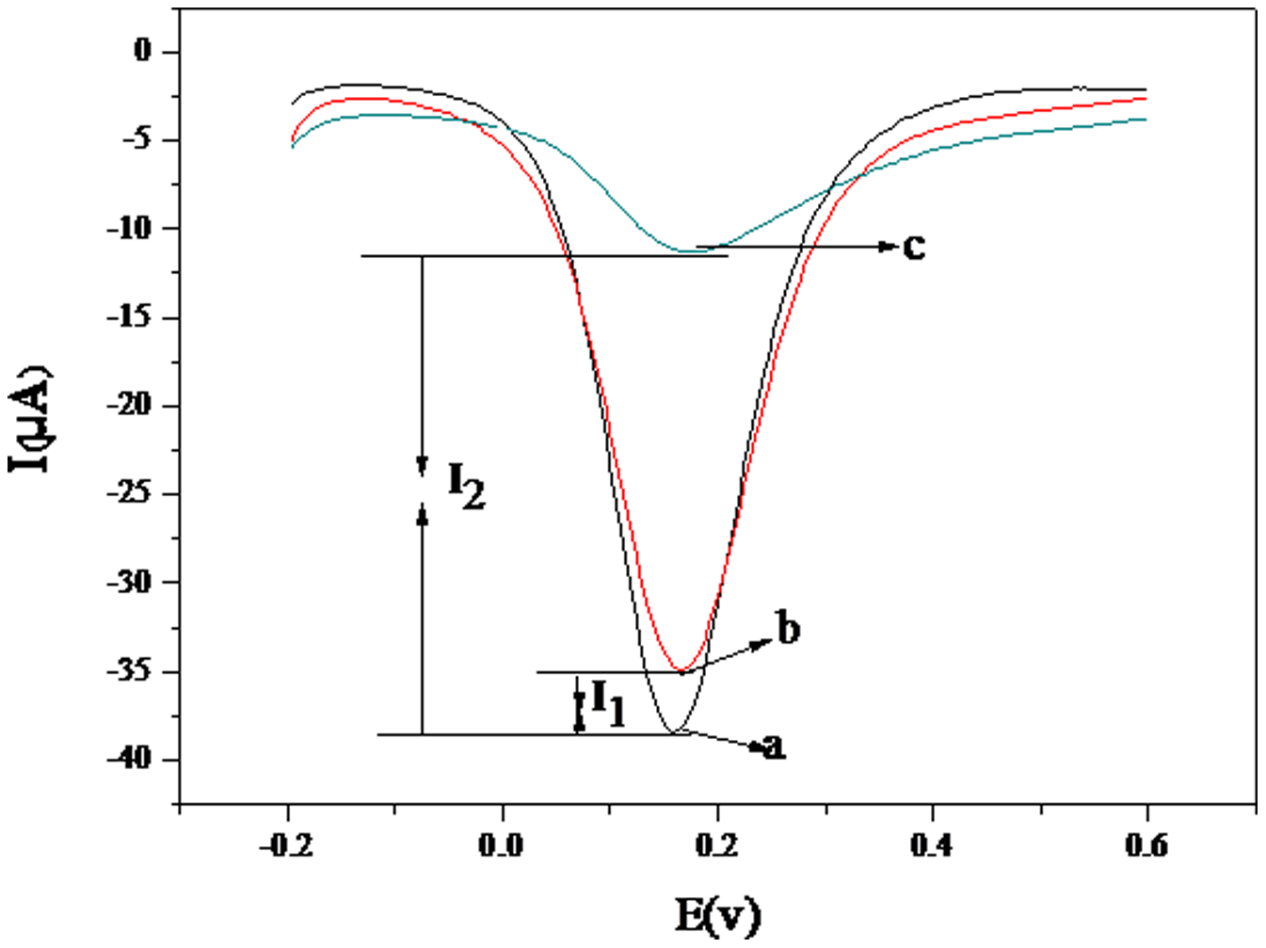
Differential pulse voltammograms of the immunosensor measurement process: (a) BSA-MAb-AuNPs–thiourea-GE; (b) H5-BSA-MAb-AuNPs-thiourea-GE; (c) GO-PAb-BSA-H5-BSA-MAb-AuNPs-thiourea-GE. Supporting electrolyte was 5(CN)_6_^3−/4−^, 0.1 M KCl and 0.01 M PBS (pH 7.0).

The signal amplification was also confirmed by differential pulse voltammetry (DPV) measurements. As shown in Figure 4, a 7-fold increase in the change current was observed with GO-PAb-BSA-H5-BSA-MAb-AuNPs-thiourea-GE (I_2_) compared with H5-BSA-MAb-AuNPs–thiourea-GE (I_1_). This can be explained as GO-PAb-BSA introduced more proteins onto the electrode surface, preventing further transfer from the redox probe to the electrode surface.

### Optimization of analytical conditions

**Figure 5:**
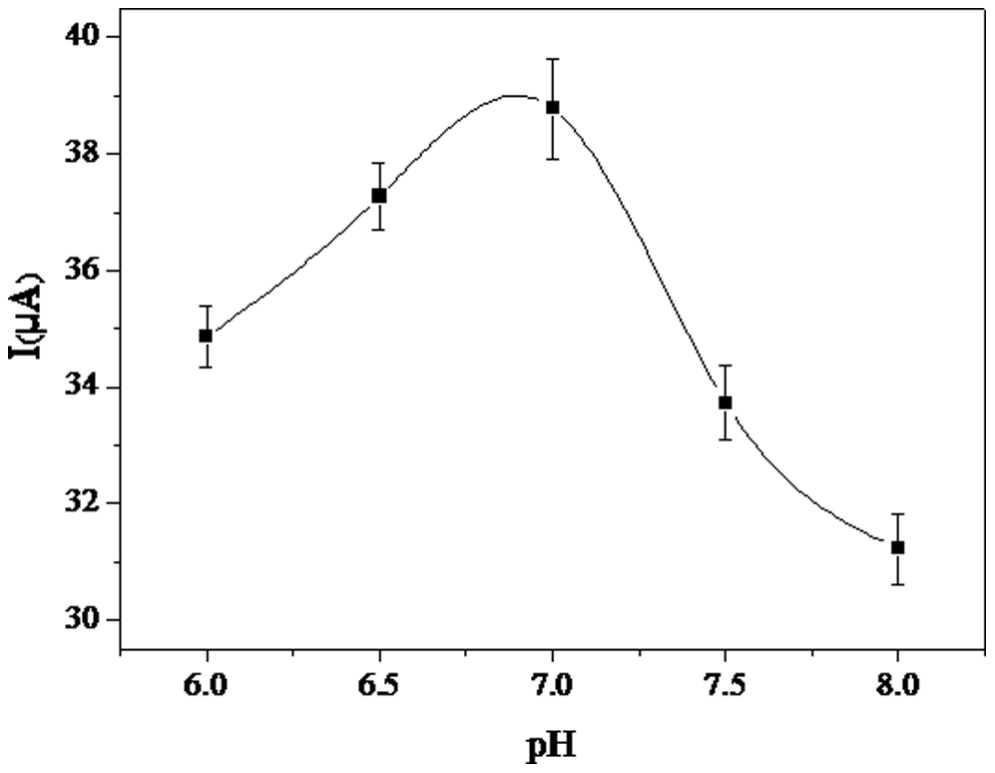
The effect of pH on the current response of the immunosensor (in 5 mM Fe(CN)_6_^4−/3−^ solution containing 0.1 M KCl). The scan rate was 50·s^−1^. Initial potential and the end potential were 0.6 V and −0.2 V, respectively.

The effect of pH on the immunosensor was investigated between pH 6.0 and pH 8.0. As shown in Figure 5, increasing the pH from 6.0 to 7.0 resulted in an increased peak current; further increases in pH resulted in the peak current decreasing. These results showed that the maximum current response occurred at pH 7.0. Therefore, PBS at pH 7.0 was used throughout this study.

**Figure 6:**
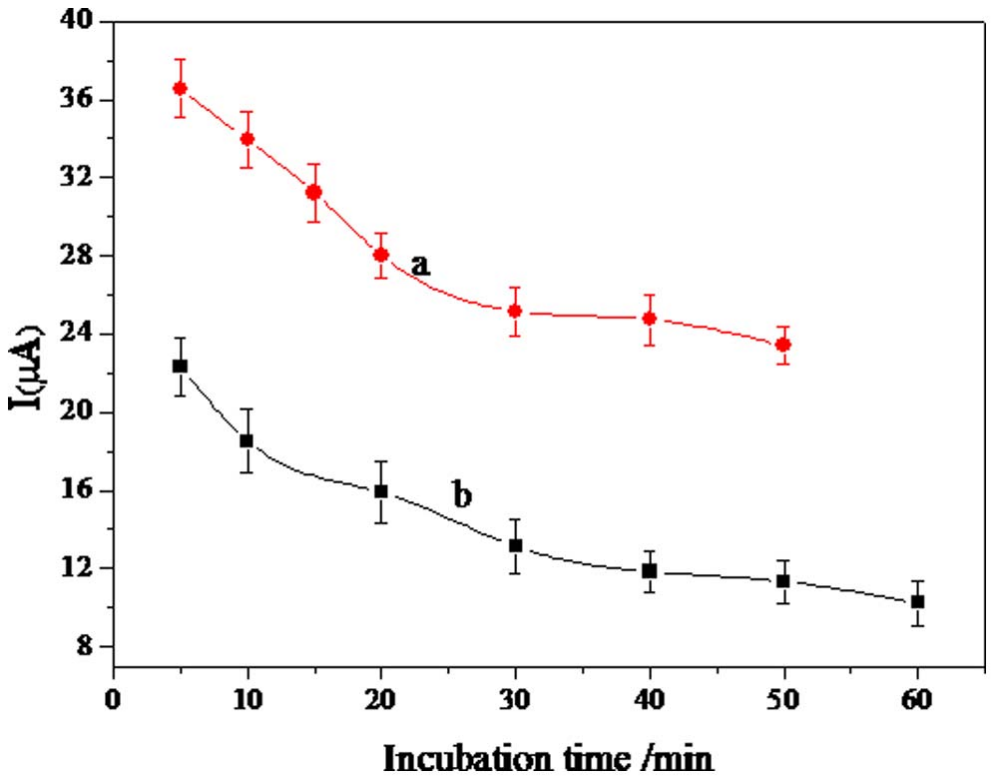
Influence of the incubation time on the current response (a) H5 (b) GO-PAb-BSA.

The incubation time is an important parameter for both capturing H5 antigen and specifically recognizing GO-PAb-BSA. We showed that the electrochemical response decreased with increasing H5 antigen incubation time and tended to reach a steady value after 30 min (curve a, Figure 6), indicating thorough capture of the antigens on the electrode surface. In the second immunoassay incubation step, the current also decreased upon increasing incubation time and it reached a plateau at 40 min, which indicates that binding sites between the antigen and detecting antibody were saturated (curve b, Figure 6).

### Criteria for judgement of positive or negative samples

According to the principle of statistics, if the current of a sample is more than the critical value (critical value  =  average current of the negative sample +3×standard deviation), the sample is considered as positive. Eighteen samples were tested negative by immunosensor (the average current: −38.46, standard deviation: 0.38), the critical value is −37.32. When a current of a sample is more than −37.32, the sample will be considered positive for the H5 antigen.

### Performance of immunosensor

**Figure 7:**
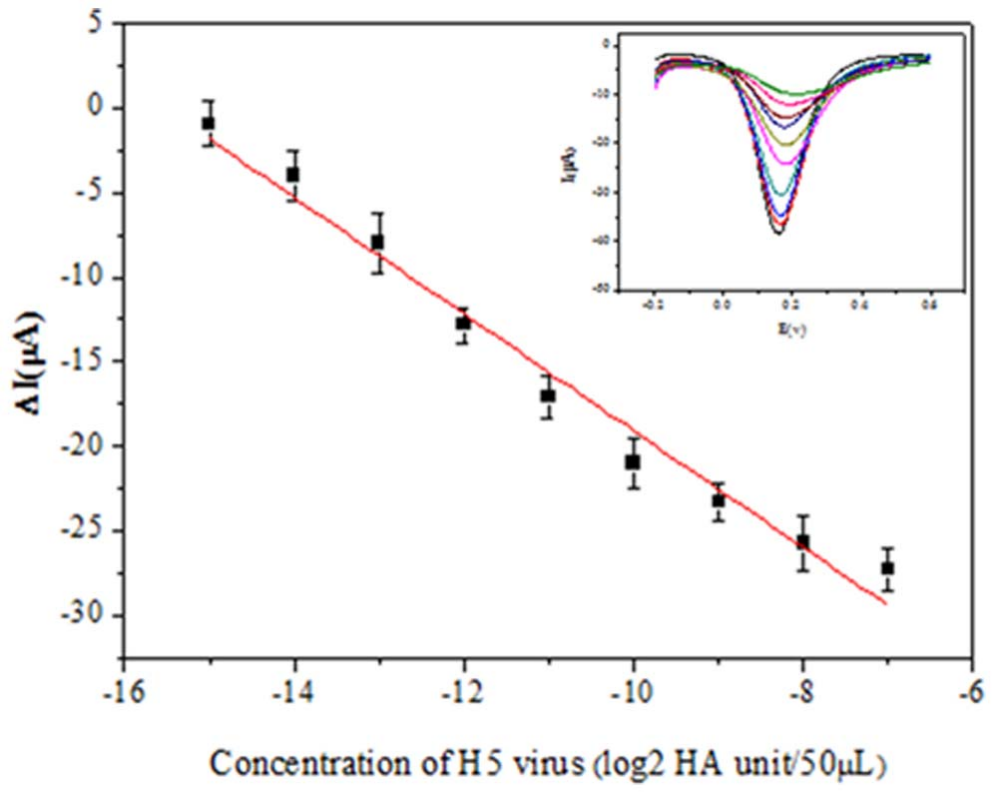
The relationship between antigen concentrations and the sensor response to current. Inset: DPV of the GO-PAb-BSA modified immunosensor with various concentrations of H5 (from top to bottom: 0, 2^−15^, 2^−14^, 2^−13^, 2^−12^, 2^−11^, 2^−10^, 2^−9^, 2^−8^, and 2^−7^ HA unit/50 µL, in pH 7.0 PBS containing 5 mM K_4_Fe(CN)_6_, 5 mM K_3_Fe(CN)_6_ and 0.1 M KCl).

Immunosensor performance was evaluated according to its ability to detect H5 using the DPV technique (in 5 mM Fe(CN)_6_^3−/4−^, 0.1 M KCl and 0.01 M PBS), under optimized sandwich-type immunoreaction conditions. As expected for a sandwich mechanism, the DPV peak current density of the immunosensor decreased with increasing H5 concentrations. Figure 7 illustrates the calibration plots of the cathodic peak current in response to varying H5 concentrations. According to the amplification effect of the GO-PAb-BSA, the linear range spans H5 concentrations of 2^−15^ to 2^−8^ HA unit/50 µL with a detection limit of 2^−15^ HA unit/50 µL (Figure 7). For comparison, the current response of the immunosensor was also recorded without GO-PAb-BSA amplification. In the absence of GO-PAb-BSA amplification, the linear range spans H5 concentrations from 2^−7^ to 2^0^ HA unit/50 µL.

### Specificity study

**Figure 8:**
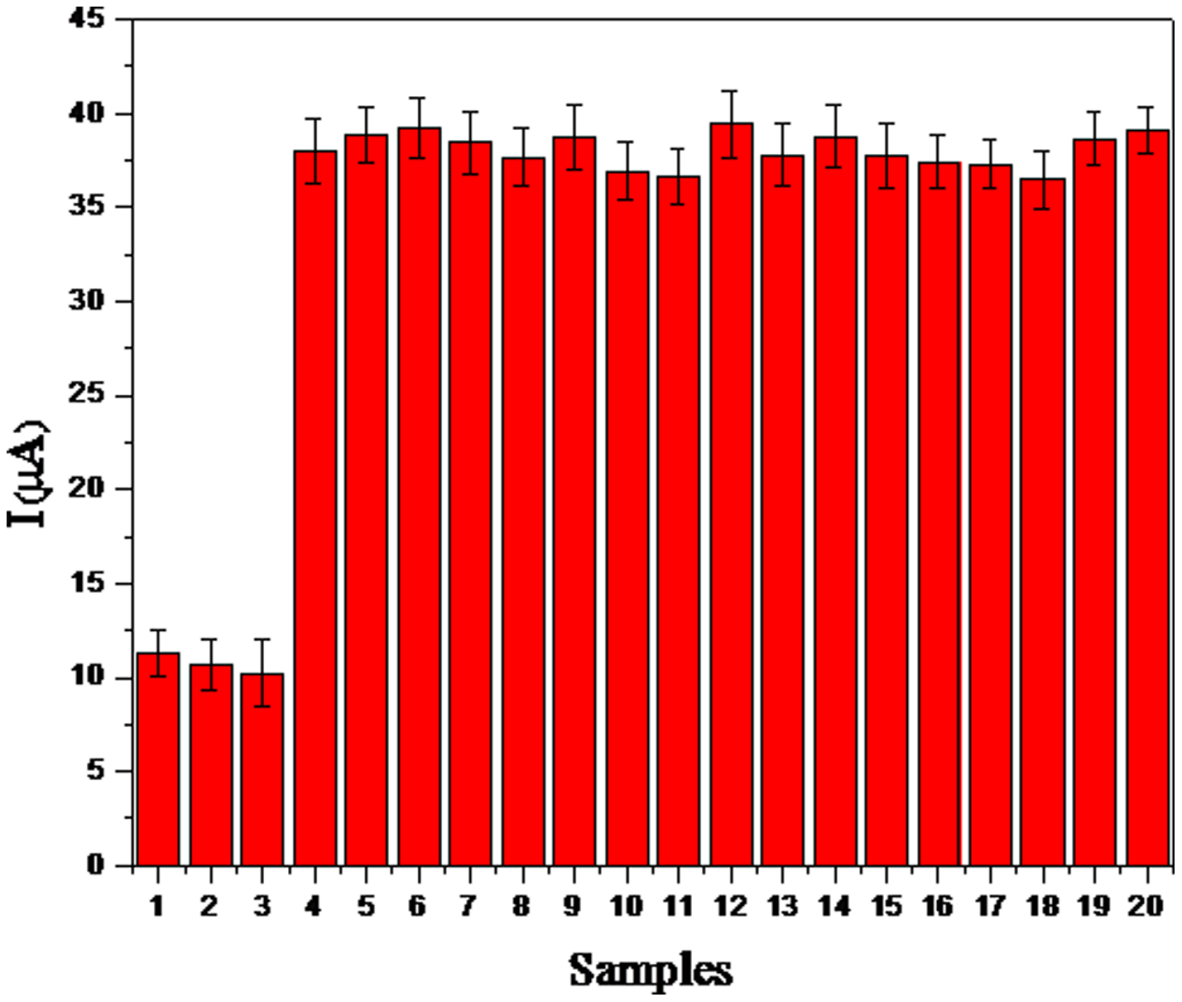
Selectivity of the electrochemical immunosensor: (1) H5N1; (2) H5N2; (3) H5N9; (4) H1N3; (5) H2N3; (6) H3N6; (7) H4N5; (8) H6N8; (9) H7N2; (10) H8N4; (11) H9N6; (12) H10N3; (13) H11N3; (14) H12N5; (15) H13N5; (16) NDV; (17) IBV; (18) ILTV; (19) MG; (20) SPF chicken.

We tested our developed immunosensor against some non-target samples such as H1N3, H2N3, H3N6, H4N5, H6N8, H7N2, H8N4, H9N6, H10N3, H11N3, H12N5, H13N5, NDV, ILTV, IBV and MG. The experimental procedure was the same as that used for the H5 target. Figure 8 demonstrates that all the non-target samples produced baseline signals similar to the negative control, tissues from the SPF chickens. The results indicate that our developed immunosensor has good specificity for the target H5.

## Discussion

Electrochemical immunosensors have been proven as an inexpensive and simple analytical method with remarkable detection sensitivity, and ease of miniaturization [26]. Various types of electrochemical immunosensors have been reported, including, amperometric, potentiometric, capacitive and impedance immunosensors. Amperometric electrochemical immunosensors have been considered as one of the most potential approaches for a higher sensitivity, less complicated instrumentation and broad linear range [27]. Therefore, in this study we investigated an amperometric electrochemical immunosensor for detecting AIV H5.

Because of the large specific surface area, high surface free energy, good biocompatibility and suitability, many kinds of nanomaterials have been widely used in electrochemical immunosensors, including metal nanoparticles (gold, silver), semiconductor nanoparticles and electroactive component-loaded nanovehicles (silica nanoparticle, polymer beads, and liposome beads). Compared to the traditional metal ion labels, enzyme labels and redox probe labels, the common characteristic of nanomaterial labels in electrochemical immunosensors lies in their ability to provide signal amplification [28].

For our immunosensor fabrication, thiourea was self-assembled on a gold electrode (GE) via Au–S covalent bonds, which then yielded an interface containing amine groups ready for the electrochemical reduction of HAuCl_4_, preparation of AuNP-modified GE surfaces and H5 monoclonal antibody attachment. AuNPs were used to promote electron transfer between proteins and electrodes. In particular, AuNPs have been extensively used as an immobilizing matrix for retaining the bioactivity of antibodies.

Our present work highlights the promising applications of BSA functionalized GO in signal amplification for ultrasensitive detection of AIV H5. Herein, GO was employed as a nanocarrier for BSA and antibody co-immobilization. Enhanced sensitivity for the AIV H5 was based on the following signal amplification strategy: first, the high specific surface area of GO allowed multiplel binding events of BSA and second, GO and BSA hindered the diffusion of ferricyanide toward the electrode surface.

## Conclusions

In summary, we have successfully designed a PAb labeled GO immunosensor and demonstrated its use in the ultrasensitive and selective detection of H5. Enhanced sensitivity was achieved by using GO as a nanocarrier to link BSA and PAb at a high ratio. Our immunosensor detected H5 antigen efficiently over a broad linear range and with a high sensitivity. We anticipate that this method may be extended for determination of other proteins and may have a promising potential in clinical applications.
